# MicroRNA Signatures in the Upper Urinary Tract Urothelial Carcinoma Scenario: Ready for the Game Changer?

**DOI:** 10.3390/ijms23052602

**Published:** 2022-02-26

**Authors:** Alessandra Cinque, Anna Capasso, Riccardo Vago, Matteo Floris, Michael W. Lee, Roberto Minnei, Francesco Trevisani

**Affiliations:** 1 Biorek S.r.l., San Raffaele Scientific Institute, 20132 Milan, Italy; alessandra.cinque@biorek.eu; 2 Department of Medical Oncology Livestrong Cancer Institutes, Dell Medical School, University of Texas at Austin, Austin, TX 78723, USA; anna.capasso@austin.utexas.edu; 3 Urological Research Institute, Division of Experimental Oncology, IRCCS San Raffaele Scientific Institute, 20132 Milan, Italy; vago.riccardo@hsr.it; 4 Faculty of Medicine and Surgery,, Università Vita-Salute San Raffaele, 20132 Milan, Italy; 5 Nephrology, Dialysis, and Transplantation, Università degli Studi di Cagliari, G. Brotzu Hospital, 09134 Cagliari, Italy; matteo.floris@aob.it (M.F.); rob.minnei@gmail.com (R.M.); 6 Department of Medical Oncology and Medical Education, Dell Medical School, Livestrong Cancer Institutes, University of Texas at Austin, Austin, TX 78723, USA; lee_michael@austin.utexas.edu; 7 Unit of Urology, San Raffaele Scientific Institute, 20132 Milan, Italy

**Keywords:** upper urinary tract urothelial carcinoma, biomarkers, microRNAs

## Abstract

Upper urinary tract urothelial carcinoma (UTUC) represents a minor subgroup of malignancies arising in the urothelium of the renal pelvis or ureter. The estimated annual incidence is around 2 cases per 100,000 people, with a mean age at diagnosis of 73 years. UTUC is more frequently diagnosed in an invasive or metastatic stage. However, even though the incidence of UTUC is not high, UTUC tends to be aggressive and rapidly progressing with a poor prognosis in some patients. A significant challenge in UTUC is ensuring accurate and timely diagnosis, which is complicated by the non-specific nature of symptoms seen at the onset of disease. Moreover, there is a lack of biomarkers capable of identifying the early presence of the malignancy and guide-tailored medical treatment. However, the growing understanding of the molecular biology underlying UTUC has led to the discovery of promising new biomarkers. Among these biomarkers, there is a class of small non-coding RNA biomarkers known as microRNAs (miRNAs) that are particularly promising. In this review, we will analyze the main characteristics of UTUC and focus on microRNAs as possible novel tools that could enter clinical practice in order to optimize the current diagnostic and prognostic algorithm.

## 1. Introduction

Upper urinary tract urothelial carcinoma (UTUC) is a minor subgroup of malignancies arising in the urothelium of the renal pelvis or ureter (5–10%) [1]. The estimated annual incidence is around two cases per 100,000 people [2], with a mean age at diagnosis of 73 years, and a male predominance of 2–3:1 is reported [2,3].

UTUC can be multifocal and/or metachronous, with an underlying oligoclonality or, more often, monoclonality [4]. In 17% of UTUC cases, a concurrent bladder cancer is present [5], while a recurrence in the bladder or in the contralateral upper tract is observed in 4.6–47% and in 2–6% of cases, respectively [6,7,8,9].

## 2. Risk Factors and Genetics of UTUCs

UTUCs are usually sporadic, though familial/hereditary UTUCs may be observed. Indeed, up to 20% of familial/hereditary UTUC cases seem to be related to Lynch’s syndrome (LS), which is a hereditary non-polyposis colorectal carcinoma (HNPCC)-related tumor [10] that is characterized by a female predominance, younger age at onset [11] and a greater susceptibility to developing a contralateral UTUC [12]. The European Association of Urology (EAU) UTUC guidelines (GL) provide a weak recommendation on evaluation based on the Amsterdam criteria, in patients suspected of having LS, to identify individuals and families at risk for HNPCC-related tumors [13].

Beyond hereditary and genetic risk-associated factors, environmental factors such as smoking and, in certain geographical areas, aristolochic acid are strongly linked to sporadic UTUCs. This supports a causative role for carcinogenic agents [13] especially in individuals who are more susceptible to UTUC. Both tobacco product use and exposure to aristolochic acid should be assessed as risk factors according to the 2021 EAU guidelines for upper tract urothelial cancer (weak recommendation) [14]. Indeed, aristolochic acid is a recently investigated, potential carcinogenic agent, known to cause Balkan endemic nephropathy/Chinese herb nephropathy, and to induce a p53 gene mutation, thus resulting in a higher regional UTUC risk [15]. For instance, the incidence in Taiwan is so high that one-fourth of all UTUCs arise from the upper urothelial tract [16], whereas tobacco use, on the other hand, is a known risk factor for UTUC with a relative risk (RR) of 2.5–7 [17]. An association with worse oncological outcome after a radical nephroureterectomy has been observed, with an increased risk of UC recurrence and mortality [18]. Moreover, UTUC are associated with Black foot vasculitis and CYC treatment [17].

## 3. Diagnosis and Staging

UTUCs are more frequently diagnosed in an invasive or metastatic stage, comprising approximately 60% and 7% of total cases, respectively [15]. As a consequence, the diagnosis of UTUC is often incidental or discovered upon further evaluation prompted by clinical manifestations due to local or systemic disease [19]. The most common presenting symptoms include, in order of their prevalence, visible or non-visible hematuria (73–80%) [20,21], flank pain (30%), or a palpable mass, the latter of which is infrequently observed (6%) [21]. Patients with UTUC who develop systemic symptoms require an appropriate evaluation to assess the presence, site and number of metastases, all of which are associated with a worse prognosis [19].

A variety of diagnostic modalities are employed to facilitate both the detection and staging of UTUC and multifocal UC, as well as more complete risk stratification and prognostic evaluation in the pre-operative setting. This, in turn, supports a multidisciplinary team-decision making process focused on making the most appropriate decisions on optimal treatment strategies [15].

In patients suspected of having an UTUC, whether arising from an unexpected incidental lesion detected during an imaging procedure or following an initial evaluation for hematuria and/or flank pain, the first-choice modality for diagnosis and staging is computed tomography urography (CT-U), according to the EAU UTUC guidelines [14]. Of available imaging techniques, CT-U has the highest diagnostic accuracy with a greater than 90% sensitivity and specificity (92–97% and 93–95%, respectively) [22,23], a 79% positive predictive value and a high negative predictive value (99%) [22]. Furthermore, imaging findings, such as more severe hydronephrosis or CT-texture analysis, seem to predict non-organ-confined disease, although the use of CT-U in risk stratification needs further study to be validated for clinical practice [24].

An alternative to CT-U is magnetic resonance urography [25], though its use is only weakly recommended and it must be used with caution in patients with category G4 or G5 chronic kidney disease (CKD) [14]. Nonetheless, relative safety in this context has been observed; however, there is the risk of systemic nephrogenic fibrosis when receiving one dose of group II gadolinium-based contrast administration below 0.07% in CKD G4–G5 patients, below 0.5% in CKD G5 non-dialysis subjects, and below 0.2% for dialysis-dependent patients [26].

An integral part of the uro-oncologic work-up is the use of urethrocystoscopy to identify or exclude concomitant bladder cancer, since UCs are often multifocal. The aim is to achieve precise disease staging and early diagnosis of multifocal UC in order to tailor treatment to individual patients [5,15].

Another useful diagnostic tool is cytology, although it is only modestly predictive for high grade or muscle-invasive UC in patients who have already undergone a radical surgery. Therefore, it should only be performed selectively for the affected upper tract [27] and it should be integrated with other endoscopic and imaging data.

Indeed, positive or suspicious cytology findings require in-depth evaluation and close follow-up even when endoscopy is negative [28]. As a matter of fact, abnormal cytologic findings may suggest a high-grade UTUC even when a bladder lesion is not identified by cystoscopy, and is considered a significative pre-operative variable in UTUC risk-stratification. Moreover, an abnormal cytology examination may be observed in bladder or prostatic-urethra in situ carcinomas undetected by endoscopic examination (e.g., hexaminolevulinate-guided fluorescence cystoscopy) [28,29] or other additional diagnostic techniques (e.g., fluorescence in situ hybridization) [28].

Diagnostic flexible ureterorenoscopy (URS) allows direct endoscopic visualization of visible lesions in the upper urinary tract through white light endoscopy [15,30]. Moreover, growing evidence on the use of techniques such as narrow-band imaging or photodynamic diagnostic flexible URS support a possible role in the detection of flat lesions that would otherwise be overlooked [31,32]. The other advantage of URS is its ability to perform not only cytologic but also biopsy sampling of any identified suspicious lesions, although tumor under-grading may occur and it is unreliable for pre-RNU staging purposes, with a 63% overall accuracy (when used as the sole staging technique) [33], since lamina propria is often not included in URS biopsies (in up to 32% of cases). Furthermore, the accuracy of histopathological examination of URS bioptic sampling can be limited by inadequate tissue-sampling artefacts. On the other hand, URS biopsy grade appears to correlate with pathological stage and is considered a remarkable pre-operative variable in risk stratification [30]. In summation, URS is not an upfront diagnostic-staging technique and should be used in the setting of insufficient diagnostic and risk stratification data collected by UCT, cystoscopy and cytology [30,34].

CT of the chest, abdomen and pelvis is performed as a means to detect distant metastases, allowing thorough staging of UTUC, and consequently directly guiding treatment planning in case of metastatic or non-metastatic disease [14]. Encouraging, but preliminary data on FDG-PET/CT staging are emerging, with 85% sensitivity in detecting metastases, nonetheless insufficient to support its indication in clinical practice guidelines at the moment [35].

UTUC can be stratified according to stage as non-invasive papillary tumors, carcinoma in situ and invasive carcinomas, [36] (Herrera Puerto et al., 1989) the latter being the most common scenario observed at diagnosis and staging should be expressed according to the 8th edition of tumor, node, metastasis (TNM) classification [14].

In summary, distinguishing a non-muscle invasive from a muscle-invasive UTUC can be challenging in the pre-operatory setting, since URS is not a reliable staging tool. For this reason, a number of pre-operative prognostic factors, predictive tools such as pre-radical nephroureterectomy models and prognostic nomograms have been evaluated to risk stratify UTUC, with the aim of differentiating between organ-confined and non-organ confined/muscle-invasive UTUC and select those who can benefit the most from a non-radical surgical approach [34].

## 4. Classification, Prognosis and Risk-Stratification

The histological classification and grading of UTUCs are based on the WHO classification, since most of the published data still use the 1973 WHO classification [37]. UTUC can also present with a wide spectrum of histologic variants, introducing further prognostic variability as they are well-recognized postoperative negative prognostic factors [38].

UTUCs are often diagnosed as invasive cancer and prognosis is strongly influenced by the TNM stage. In a series, 5-year overall survival was 73%; for pT0, pTa and pTis it was 94%, while for invasive stages it dropped to 75%, 54% and 12% for pT2, pT3 and pT4, respectively [39].

The latest EAU UTUC guidelines suggest, as a weak recommendation because of the lack of level 1 supporting evidence, risk stratifying UTUC using preoperative factors for therapeutic guidance, identifying low- and high-risk cancers, the former being more likely to benefit from kidney treatment, the latter from a radical surgical approach. Moreover, chronological age alone should not preclude RNU with curative intent [14].

A wide array of prognostic factors have been investigated [40]. They are usually divided as pre-operative and post-operative, the former being of relevance in risk-stratification and in surgery planning, and for this reason are often incorporated in predictive tools used to identify non-organ-confined high-risk UTUC [14].

Before surgery, the presence of several significant prognostic factors can be taken into account by the clinician, such as a high-grade URS biopsy finding, positive or high-grade cytology findings older age [30,41,42], smoking [18,43], ureteral location and multifocality [43,44,45], locally advanced disease with hydronephrosis [46,47], a surgical delay over 12 weeks [48,49], systemic symptoms suggestive for advanced-metastatic disease [14] obesity and higher body mass index [50] high pre-treatment-derived neutrophil–lymphocyte ratio [51,52].

The most important intra- and post-operative prognostic factors reported in the literature are primarily tumor stage and gradewhile other relevant factors include lymph node (LN) involvement and extra-nodal extension [53], lymph vascular invasion, positive surgical margins [54,55], variant histology, extensive tumor necrosis [56,57,58], sessile growth pattern [59,60] concomitant CIS in organ-confined UTUC, history of bladder CIS [59,61] and distal ureteral management [14].

As in bladder cancer, a wide spectrum of molecular markers (related to cell adhesion, microsatellite instability, cell differentiation, angiogenesis, cell proliferation, epithelial-mesenchymal transition, mitosis, apoptosis, vascular invasion, programmed death-ligand 1 expression, c-MET), including miRNA, have been studied. Nevertheless, there is still insufficient evidence of their prognostic impact to support their use in clinical practice, since at present their use is not clinically validated [14,40].

## 5. Treatment Management

Treatment decision-making is guided by prognostic evaluation using tumor stage. As a matter of fact, non-metastatic and metastatic UTUCs require different management with different treatment goals: potentially curative and palliative, respectively [34,54,56,62].

A non-metastatic UTUC can be risk stratified as a low-risk carcinoma (unifocal disease, tumor size < 2 cm, low-grade cytology, low-grade URS biopsy, a non-invasive aspect on CT urography) or as a high-risk carcinoma (multifocal disease, tumor size ≥ 2 cm, hydronephrosis, high-grade cytology, high-grade URS biopsy, variant histology, prior radical cystectomy for high-grade bladder cancer) [34,63,64] 

Low-risk carcinomas are treated with kidney-sparing surgery as survival rates are similar to those after RNU [65]. Depending on the tumor characteristics, the clinician may choose endoscopic ablation with flexible ureteroscopy [66,67], segmental resection or a percutaneous approach [65,68].

Low-level evidence supports the use of upper urinary tract instillation of Bacillus Calmette–Guerin (BCG) or mitomycin C [69,70], performed either with an anterograde, retrograde or combined approach [71,72,73], since both treatment modalities result in similar overall survival, recurrence and progression rates [74].

The standard of care for high-risk carcinomas is RNU, with an open [39], laparoscopic [75,76] or robotic approach [77,78]. On the contrary, in patients with a solitary kidney and/or impaired renal function, kidney-sparing management should be considered on a case-by-case basis [79]. Along with the radical resection of the tumor, the homolateral kidney and ureter, the patient is offered a bladder cuff resection, which consists in a vesical ureteral orifice resection, to further reduce the risk of bladder UC recurrence [80,81,82].

Open RNU, laparoscopic and robotic approaches all have similar oncological outcomes in patients with organ-confined UTUCs, while for non-organ-confined cancers (cT3/4, cN+, cM+ in the TNM classification) an open RNU approach is the preferred choice [83,84,85,86,87].

In the setting of high risk UTUC already in the muscle-invasive stage, also performing a lymph node dissection (LND) is associated with lower recurrence rates, improved cancer-specific survival [88], and improved survival regardless of the “N” stage [89,90,91]. Two to ten days after RNU, the intravesical instillation of a single dose of BCG or Mitomycin C is associated with reduced bladder UC recurrence rate [92,93]. EAU GLs for UTUC also suggest, with a weak strength recommendation, intravesical chemotherapeutic instillation in the setting of kidney-sparing surgery [14].

The role of neoadjuvant chemotherapy (NeoCT) has been evaluated in patients with advanced UTUC; despite the lack of randomized controlled trials, contemporary literature is rapidly growing and has also been recently enriched with prospective evidence that led it to be considered as a favorable option. NeoCT usually includes a platinum compound and has been associated with lower pathological downstaging: >60% of high-grade patients showed a ≤1 ypT1 stage after neoadjuvant therapy in a phase II trial. Furthermore, a recent metanalysis observed a 38% pooled pathologic tumor downstaging rate. Moreover, NeoCT increased complete response rates (especially in high-grade UTUC, in up to 14% of cases), and lower disease recurrence and mortality rates versus standard radical surgery [34,64].

Adjuvant chemotherapy is an option for patients with locally advanced UTUC, though controversies remain based on the conflicting results reported in the current literature [94,95,96,97] and the greater susceptibility to chemotherapy-induced kidney injury in patients with an iatrogenic solitary kidney [98,99]. Current evidence supports the use of a platinum-based regimen over non-platinum regimens. UTUC-specific randomized clinical trials (RCTs) are scarce and generally support the use of an early combination of a platinum compound with another antineoplastic agent (e.g., Cisplatin + Gemcitabine) [100]. EAU GLs strongly recommend postoperative systemic chemotherapy with platinum-based regimens in patients with muscle-invasive UTUC [14].

Metastatic UTUC treatment is primarily based on systemic chemotherapy as a palliative treatment [34,62,101]. Even in the presence of metastases, there is room for some surgical options. As a matter of fact, RNU might be offered to symptomatic patients with resectable, locally advanced disease, as it may improve quality of life and outcomes [102,103,104], gaining a “weak recommendation” in the EAU UTUC guidelines if a resection is still technically feasible [14].

Metastasectomy is an option, although the absence of evidence supporting its efficacy imposes a case-by-case evaluation with the patient [14]. Current UTUC-specific evidence on systemic chemotherapy is growing, though bladder-cancer-related literature still plays a big role in supporting chemotherapy practices in UTUC patients. Chemotherapy should be offered to all patients with metastatic disease since cisplatin-based regimens can improve median survival, while single-agent and carboplatin-based combinations are less effective than cisplatin-based combination chemotherapy in terms of complete response and survival [62,64,101,105].

Platinum-based combination chemotherapy is the treatment of choice in the first-line management of cisplatin-eligible patients [62,64,101]. Cisplatin can be combined with Gemcitabine (GC) or Methotrexate + Vinblastine + Adriamycin (MVAC and high-dose MVAC) or Paclitaxel + Gemcitabine (PCG) and the use of these regimens is strongly recommended by the EAU GLs [14].

Carboplatin might be a first-choice agent in cisplatin-ineligible patients, coupled with another antineoplastic agent in a carboplatin-based regimen [106]. In the first line setting, two immune checkpoint inhibitors (ICIs) have been approved for UTUC management. Currently, pembrolizumab [107] and atezolizumab [108,109] have been approved by the FDA and EMA and are clinically available, though the EAU GLs weakly support their use in this setting due to the scarce, but growing, literature [14].

If a patient treated in the first line setting displays progression during or after administration of chemotherapy or ICIs, several second-line agents can be used, though the EAU GLs strongly recommend only pembrolizumab, atezolizumab or nivolumab, while they state that vinflunine monotherapy should be offered as a third or subsequent treatment line or as second-line treatment if immune checkpoint inhibitors or combination chemotherapy are not feasible [14].

At the moment, different ICIs have been approved by the FDA and EMA for metastatic UTUC-patients in the second-line setting, as they share similar efficacy and safety, though UTUC-specific literature is scarce. Pembrolizumab and Atezolizumab can also be used in this context [110,111,112,113], whereas Avelumab, Durvalumab and Nivolumab have only been approved as second-line agents [114,115,116,117].

Given the wide array of treatment-related toxicities, as with other antineoplastic agents, UTUC-patients treated with ICIs require multidisciplinary care [118].

Management of UTUCs remains difficult due to the limitations of the current predictive and prognostic tools. Therefore, specific biomarkers must be identified to predict outcomes and tailor personalized treatment and surveillance strategies in order to increase survival and decrease morbidity in UTUC affected patients.

## 6. MicroRNAs as Biomarkers in UTUC: Proof in Principle

The growing understanding of the molecular biology underlying UTUC has led to the discovery of promising new biomarkers. Among the most promising is a class of small non-coding RNA biomarkers known as microRNAs (miRNAs).

miRNAs are relatively small (approximately 18–24 nucleotides), single-stranded endogenous RNA molecules that negatively regulate gene expression at the post-transcriptional level by binding to the 3′-untranslated region of target messenger RNA (mRNA) [119,120]. They can repress translation or lead to degradation of mRNA targets based on imperfect or perfect complementarity between the miRNA and the mRNA sequence, respectively [119]. According to the latest release of the miRBase database (v.22), there are now more than 2600 unique mature human miRNAs [121]. A single miRNA can regulate up to 200 mRNA transcripts [122], and thousands of human genes are conserved targets of miRNAs [123]. Given this vast majority of mRNA targets regulated by miRNAs, miRNAs potentially influence almost all genetic pathways [122]. Indeed, miRNAs play a crucial role in diverse biological processes including development, cell growth, differentiation, apoptosis and proliferation [124]. The dysregulation of these processes is a hallmark of cancer [125]. As might be expected, an altered expression of miRNAs has been associated with the pathogenesis of cancer, including initiation and progression of cancer, as well as with many other pathological conditions [126].

It has been estimated that more than half of the miRNA genes are located in cancer-associated genomic regions or in fragile sites [127]. Based on the role of their mRNA targets, deregulated miRNAs can act as oncogenes (oncomiRs) or tumor-suppressor genes (tumor suppressive miRs) [128]. OncomiRs are mostly overexpressed and tumor suppressive miRs are under-expressed in cancer [126].

Aberrant expression levels of miRNA transcripts, either downregulated or upregulated, in comparison with those in the corresponding normal tissues have been observed in a broad variety of human malignant cancers [129,130,131,132,133,134,135,136,137,138]. miRNA expression profiles can be unique for different cancer types and may also characterize tumor histology. In addition, miRNA expression can change during tumor progression, reflecting the clinicopathological features of the tumor such as grade, stage, aggressiveness, vascular invasion and proliferation index [128,139]. Thus, miRNAs could be ideal candidates as diagnostic and prognostic biomarkers. miRNAs could also be involved in chemoresistance, as has been shown in many studies [128,140,141,142,143,144,145,146,147,148], and consequently could also be used as predictive biomarkers as well as constituting possible therapeutic targets.

miRNAs are not only present in tissue cells but can also be released into the extracellular space and then transported into bodily fluids, such as peripheral blood and urine. These cell-free or circulating miRNAs are key regulators of cellular crosstalk, modulating gene expression in recipient cells under normal and pathological conditions, such as cancer. Indeed, as part of a cell-to-cell communication mechanism in cancer, circulating miRNAs have been associated with cell proliferation and migration, metastasis, epithelial–mesenchymal transition (EMT), angiogenesis and evasion of immune response [149,150]. miRNAs can be released by cells through both vesicle trafficking and protein carrier mechanisms: (i) packaged into extracellular vesicles (EVs), such as exosomes, microvescicles, or apoptotic bodies; (ii) associated with high-density lipoprotein (HDL); (iii) or associated with RNA-binding proteins such as argonaute proteins (Ago1 or Ago2), nucleophosmin (NPM1), or ribosomal proteins [151,152,153,154,155].

miRNAs are thus also protected from degradation by RNase and are very stable in biofluids. Recipient cells can then uptake EVs carrying miRNAs via different mechanisms, including direct membrane fusion, endocytosis, micropinocytosis, phagocytosis and receptor binding. HDL-binding miRNAs can also be delivered to recipient cells via unknown mechanisms. On the other hand, there is no evidence of AGO2–miRNA complexes or NPM1-bound miRNAs being delivered into recipient cells [151,152,153,154,155].

Levels of circulating miRNAs in the plasma and serum of healthy individuals were found to be constant [156,157], and miRNA signatures in blood are similar in men and women and independent of the patient’s age [158,159]. On the other hand, specific expression profiles of circulating miRNAs have been shown in a variety of cancers, including bladder [160], renal [161], gastric [162], pancreatic [163], brain [164] and lung cancers [165,166] and could be used to distinguish cancer patients from healthy individuals, as well as for the prognosis of the disease.

Another characteristic that further supports miRNAs’ potential as reliable biomarkers is their high stability in biological samples as well as in stored samples, including fixed tissue, blood and other bodily fluids. Indeed, fixed tissue can provide a tremendous source of material for the discovery of cancer-related biomarkers, and it has been shown that differing formalin fixation times do not significantly influence miRNA profiles in formalin-fixed, paraffin-embedded (FFPE) specimens, and that miRNAs are well preserved even over prolonged FFPE block storage [167,168]. In the same way, circulating miRNAs are stable after being submitted to severe conditions such as boiling, extreme pH, long storage, and several freeze–thaw cycles, that would normally degrade most RNAs [156,169]. They are also protected from degradation by RNase, as explained above.

Consequently, miRNAs could be reliable biomarkers for clinical application due to (i) their altered levels during pathological processes or diseases such as cancer; (ii) their high stability in biological samples; and (iii) the highly sensitive, accurate, and reproducible measurement methods through which they can be quantified (such as quantitative polymerase chain reaction (qPCR)). Regarding circulating miRNAs, another great advantage consists of their ease of access through non-invasive or minimally invasive isolation methods.

## 7. Methods for Collecting and Measuring MicroRNAs from UTUC Patients

The rapid progression of technologies such as next-generation sequencing (NGS), microarrays and qPCR and the development of powerful bioinformatic tools has made genome-wide miRNA analysis and the consequent discovery of candidate miRNAs as diagnostic, prognostic and predictive cancer biomarkers or therapeutic targets more feasible [170].

However, miRNA profiling, especially in bodily fluids, could still be very challenging both because of the intrinsic characteristics of miRNAs (i.e., small size, GC content, high degree of sequence similarity within miRNA families, low abundance in circulation) and because of pre-analytical and analytical variables [170].

Pre-analytical variables, including sample collection, processing methods and sample quality can greatly impact the results. Regarding plasma and serum samples, the type of blood collection tube used, the time interval between blood draw and plasma/serum preparation and the different methods used for the separation of plasma and serum from whole blood are important pre-analytical factors to be considered [171,172,173]. Indeed, heparin in blood-collection tubes could inhibit PCR reaction, and procedures such as centrifugation or filtration could lead to different amounts of blood cell, cell fragment and platelet contamination in serum and plasma samples [171,172,173,174,175,176]. In addition, hemolysis must be evaluated in serum and plasma samples as it may alter circulating miRNA levels by up to 50-fold [177], even with an erythrocyte contamination as low as 0.008% [178]. It must also be taken into consideration that serum has a different miRNA profile compared to plasma due to the coagulation process [179]. Regarding urine samples, one of the main underlying reasons for variability among samples is urine concentration, which could differ significantly among individuals, at different times of the day (e.g., urine collected in the early morning is generally more concentrated than urine collected later in the day) and depending on physiological or pathological conditions. In addition, when analyzing miRNAs in cell-free urine supernatant, care should be taken at all steps of sample handling and processing to minimize cell lysis, as is the case for plasma and serum samples, because RNA levels from residual cells are several orders of magnitude higher than cell-free RNA levels. 

Therefore, considering that miRNAs are present at very low levels in bodily fluids, small variations in these pre-analytical variables may significantly alter circulating miRNA levels.

In order to obtain reliable results, it is also very important to use efficient methods of RNA extraction and cDNA synthesis to verify the RNA extraction and reverse transcription efficacy, e.g., using spike-in methods, and to adopt appropriate normalization strategies for differential expression analysis when miRNA expression data are obtained [170,180].

Among the various strategies for miRNA detection, RT-qPCR is the one with the highest sensitivity and accuracy and it is considered the gold standard technique for nucleic acid quantification, offering relatively simple and cost-efficient processing of samples and data [181]. RT-qPCR has increased throughput with the introduction of microfluidic card and array platforms, which allow for large-scale miRNA detection at the same time yet still with high specificity, sensitivity and high dynamic range. However, with such technology it is possible to detect only annotated miRNAs and only medium throughput can be reached [170]. Microarray is another technique for miRNA profiling based on nucleic acid hybridization between target molecules and their corresponding complementary probes. The methodology is high throughput, being able to analyze up to thousands of miRNAs in one assay, but only among those that are already known. It is also less expensive than amplification-based arrays, but it has lower dynamic range, sensitivity and specificity than RT-qPCR and NGS [170]. NGS is another promising quantitative technology for miRNA profiling that has the great advantage of allowing both the detection of known miRNAs and the discovery of novel miRNAs [170].

However, this technology is expensive and necessitates special equipment and expert bioinformaticians, so it cannot be considered user- and/or lab-friendly [170]. The use of different technologies and platforms may be one of the main reasons for the inconsistent results reported by many published studies regarding miRNAs as biomarkers of cancer, highlighting the importance of using standardized methods.

Besides the potential technical biases discussed above, an additional aspect to be considered regarding biomarker discovery is the intrinsic interindividual variability (e.g., genetic and epidemiologic variability) and the influence of disease-independent factors on circulating miRNAs levels (e.g., drug administration [182], smoking habits [183], diet [100,184] and physical activity [185]. Therefore, it is crucial during experimental design to assess the number of samples or participants that has to be included in the study to reach statistical power.

Generally, numerous steps of validation are required before biomarkers can be considered for clinical application. After the first screening and exploratory phase aimed at identifying candidate miRNAs as potential biomarkers in cancer, systematic evaluation of miRNA candidates must be performed to evaluate their ability to discriminate different conditions with high sensitivity and specificity in a larger and independent confirmatory study cohort. Ultimately, with standardized measurements and even larger cohorts of patients, the accuracy of the previous results must be revalidated to confirm miRNA candidates as biomarkers in the general population.

Finally, for the reason reported above regarding interindividual variability and environmental factors and considering that cancer is a complex and multifactorial disease [186], it is not surprising that, if used alone, miRNA could lack high sensitivity or specificity. On the other hand, a set of two or more miRNAs, a “miRNA signature”, either alone or in combination with clinical–pathological features, could show high discriminatory power [161,187,188,189,190,191,192] and have better potential for clinical use as biomarkers.

In this review, we summarize the most relevant findings of published research regarding miRNAs as candidate biomarkers of UTUC. 

## 8. Promising MicroRNA Biomarkers in UTUC

Although the etiology of UTUC is similar to that of bladder cancer (BC), since they both are urothelial carcinomas, their natural history is significantly different. BC is mostly (75–80%) diagnosed as a superficial tumor, while UTUC is often invasive at diagnosis and thus the prognosis is poorer [193]. Given the similarities of UTUC and BC, it is likely that they share common molecular features in the development of the tumor, related to the miRNA expression, which is likely to be similarly altered. To test this hypothesis, the expression of eleven miRNAs (miR-10a, miR-21, miR-96, miR-135, miR-141, miR-182, miR-200b, miR-205, miR-429, miR-520b, miR-1244), which were previously shown to be increased in BC, was studied in normal ureter and cancerous tissue samples of patients undergoing nephroureterectomy (47 UTUC tissue samples and 36 corresponding samples of histologically normal ureters) [194]. Most miRNAs selected resulted significantly upregulated in tumor tissues; miR-205 was overexpressed in poorly differentiated UTUC. The same analysis was then performed on circulating serum miRNAs from 44 UTUC patients and 34 controls with non-malignant urological diseases. MiR-141 confirmed an increased levels in UTUC with an AUC of 0.726 (95% confidence interval 0.609–0.843) as diagnostic marker. Finally, serum miRNA levels were associated with clinical–pathological parameters, showing that miR-10a and miR-135 were reduced in muscle-invasive UTUC (pTa/pT1 vs. pT2-4). No correlations between age, sex, metastasis or grading and circulating miRNA levels were identified [194]. Notably, circulating miR-141 was found to be elevated in multiple tumor types including prostate, colon and cervical cancer [195], and associated with the drug resistance in cancer chemotherapy [196], suggesting that it can be a useful biomarker in multiple cancer settings.

Since UTUC originates from the urothelial lining of the renal pelvis and calyces, a possible anatomic overlap with renal cell carcinoma (RCC) can occur. A correct diagnosis is critical to define proper surgery and post-surgical treatments. For this purpose, miRNA expression has been investigated to distinguish the most prevalent RCC subtypes and UTUC from the normal kidney [197]. The analysis of tissue sections derived from 24 ccRCC, 5 papRCC, 3 chRCC, 5 UTUC patients and 40 normal men showed that 434 miRNAs were significantly deregulated in cancerous versus normal tissues. In particular, miRNAs specific to each tumor entity were identified: miR-3648, miR-3656 and miR-3687 discriminated UTUC from the normal tissue with a median AUC > 0.94 [197].

UTUC can be caused also by toxic molecules found in certain industries or the environment, of which aristolochic acid (AA) is the most well-known and understood. It is related to nitrophenanthrene carboxylic acid and is a carcinogenic, mutagenic and nephrotoxic compound, which can be isolated from members of the plant family of Aristolochiaceae, whose consumption results in chronic kidney disease (CKD) and UTUC [198]. To investigate whether there is a difference in miRNA expression between AA-induced UTUC and common UTUC, samples from 20 patients/group were collected. Patients who experienced AA-induced UTUC previously received a renal transplant and were treated with a standard immunosuppressive regimen [198,199]. After discovery and validation processes, the expression of miR-488 and miR-181c was found to be significantly different between the two groups. Moreover, the expression of miR-488 was higher in stage I and II than stage III and IV tumors, while miR-181c was increased in tumors > 3 cm, even if these correlations were not validated in normal UTUC patients [199].

Hypoxia represents one of the most relevant environmental elements able to promote the metastatic process. As a matter of fact, hypoxia acts as protagonist in several different biological assets such as metabolism, angiogenesis, innate immunity and induction of stemness through the hypoxia-inducible factors (HIFs) which are the major components of hypoxia signaling pathways [200].

Human solid tumors are normally less oxygenated in comparison to the normal parenchyma from which they derived. The most prominent causes of cancer hypoxia are related to a deficiency or to an inappropriate vascularization, which can also be promoted by chronic anemia, a frequent condition that affects lots of oncology patients in clinical practice [201].

Unfortunately, due to this pathological characteristic, cancer cells tend to be more resistant to treatment, especially to radiotherapy and chemotherapy, resulting in an increased tumor metastases incidence. For these issues, the hypoxia phenomenon symbolizes a hallmark of several solid cancers, being strongly linked to malignant progression and poor clinical outcomes [202]. A recent study investigated the role of miR-210 as a possible oncogenic factor in the development and establishment of UTUC [203]. miR-210 plays a crucial role in the regulation of hypoxia-induced pathogenesis [204,205]. In fact, different lines of evidence highlighted that this biological mechanism is mediated by HIF-1α through the hypoxia-responsive factor situated on the proximal promoter of the miR-210-coding DNA. Therefore, miR-210 is usually considered as a robust HIF target [206].

Moreover, several works showed that miR-210 downregulation significantly reduced clonogenicity, migration and invasion, promoted cell apoptosis, augmented the percentage of cells in the G1 phase and diminished the percentage of cells in the S phase in vitro [203,207]. In this paper, the authors compared the expression of miR-210 between tumoral tissues affected by UTUC and paired non-cancerous urothelium [207]. The total cohort was composed of eighty-three patients with UTUC who received nephroureterectomy in a tertiary university hospital from 2013 to 2015 and the clinicopathologic data were collected by reviewing medical records. The article underlined the significant upregulation of miR-210 in each tumoral specimen with respect to the healthy adjacent noncancerous urothelium. Moreover, miR-210 was found over-expressed especially in high-stage and high-grade UTUC in comparison to low grade. MiR-210 levels could distinguish the neoplasm from the healthy parenchyma with an AUC of 0.904 (95% CI = 0.843–0.865, *p* < 0.001) and a sensitivity and specificity of 80% and 90%, respectively [207].

This finding represents a crucial point because, if appropriately validated on a larger cohort, miR-210 levels could be fundamental for the pathologists to identify precancerous lesions before histological characteristics are established. In addition, miR-210 and its targets may be possible intriguing candidates for the UTUC personalized therapy [203,207].

Another interesting perspective related to the altered expression of a miR in the UTUC scenario is proposed by Hsu WC [208]. In this work, the authors investigated the role of miR-145-5p in the regulation of ADP ribosylation factors 6 (ARF6) in the tumorigenesis of UTUC. MiR-145-5 p is a well-studied tumor suppressor microRNA, which is downregulated in many cancers [209] and it is related to poor prognosis in prostate, gastric, cervical, glioblastoma and non-small cell lung carcinoma (NSCLC) [210,211,212,213,214]. ADP-ribosylation factor 6 (ARF6), expressed widely in mammalian cells, is a small protein that is involved in several biological mechanisms, such as membrane trafficking and actin cytoskeletal rearrangement [215]. Different studies showed that the activation and high expression of ARF 6 protein are significantly associated with the invasion and metastasis of several types of cancers, such as breast cancer, pancreatic cancer, lung cancer, etc. [216].

In the UTUC panorama, the mechanism by which ARF6 modulates the migration and aggression of the cancer cells in the urothelium district remains unknown. To elucidate this aspect, the authors analyzed 114 formalin-fixed UTUC tissues and 40 paired noncancerous urothelium samples obtained from nephroureterectomy [208]. In the molecular analysis, ARF6 expression was observed to be higher in UTUC tissues than paired adjacent normal tissues whereas a reverse correlation between ARF6 and miR-145-5p was found in the neoplastic tissues [208]. In vitro experiments showed that miR-145-5 p overexpression blocked UTUC cells migration and invasion by negatively regulating ARF6 expression [208]. Further, the authors revealed that miR-145-5-p controlled tumor cell migration and invasion by suppressing the expression of Matrix Metallopeptidase 2 (MMP2), N-cadherin, Focal adhesion kinase and Matrix Metallopeptidase 7 (MMP7) [208]. However, all the above effects were reversed by ARF6. These findings suggest that miR-145-5p may suppress UTUC cell motility and invasion by targeting ARF6/MMP7 through the epithelial–mesenchymal transition [208].

In conclusion, this work underlined that the downregulation of miR-145-5p could be a biomarker of disease-free survival or cancer-specific survival in the UTUC asset; in parallel, the study hypothesized that miR-145 therapies and other ARF6-targeting inhibitors could be novel promising drugs for UTUC patients [208].

Previous works investigated the function of miR-30a-5p in the oncological asset as a tumor suppressor which is often downregulated in several cancerous tissues [217].

Mir-30a-5p normally reduces cell proliferation, migration and invasion processes [218]. Furthermore, this oncosuppressor miR represents a biomarker of focal and segmental glomerulosclerosis and drives the epithelial-to-mesenchymal transition (EMT) in colorectal, pulmonary and gastric cancers [219,220]. The EMT process plays a fundamental role in the aforementioned biological mechanisms related to cancer aggressiveness and recent articles highlighted that mir-30a-5p blocks the invasion of breast cancer cells thanks to the suppression of EMT progression [221]. 

A recent paper revealed a possible crosslink between UTUC and miR-30a-5p [222]. Three selected UTUC samples were analyzed with NGS and microarray techniques to define the most prominent miRNA signatures and their possible mRNA targets in UTUC tissues. Among all miRs signatures, miR-30a-5p was significantly downregulated in UTUC tumors compared to adjacent normal tissues. The authors further validated the different expression of the selected miR using qPCR in other 22 human samples collected from cancerous tissues of UTUC and matched adjacent normal urothelium in patients who underwent nephroureterectomy [222]. The functional role of miR-30a-5p in UTUC development was further validated using a UTUC cell line (BFTC-909) [222]. The study clearly demonstrated that miR-30a-5p expression was significantly reduced in UTUC samples in comparison to their healthy counterparts, suggesting its involvement in the proliferation and metastasis of UTUC cells [222]. Moreover, the pathway enrichment analysis also underlined the involvement of MAPK and PI3K/Akt pathways in the tumorigenesis of UTUC. Finally, the study offered the first in vitro proof that miR-30a-5p over-expression significantly augmented claudin-5 expression in UTUC BFTC-909 cells, giving the idea that miR-30a-5p was probably able to counteract EMT through interrupting the functioning of mediators in the tight junction pathway [222]. In fact, claudins are the major proteic components of tight junction structure and are responsible for the maintenance of intercellular adhesion, determining epithelial cell polarity [223]. A loss of claudins expression is strictly connected with aggressive neoplasms and high recurrence in several cancers [224].

Results of global analysis of miRNA expression patterns from patients to assess stage and prognosis of urothelial carcinoma have yielded interesting candidates worthy of further analysis. Using a miRNA array, Izquierdo et al. screened 754 miRNAs from 150 patient samples [225]. Of the total pool of miRNAs, twenty-six were found to be differentially expressed in patients with progressing or non-progressing disease. These included miRNA-31 and miRNA-149 which, upon analysis of tissue samples of 18 patients, exhibited the greatest fold change. Both miRNA-31 and miRNA-149 were, in general, expressed more highly in the non-progression group compared to the progression group. Subsequent multivariate regression analysis revealed that both of these miRNAs were independent prognostic factors of tumor progression and pathological tumor stage, whereas miRNA-149 was also an independent prognostic factor of cancer specific survival [225]. These miRNAs are aberrantly expressed in several other cancer types (i.e., clear-cell RCC, squamous cell carcinoma of the tongue, prostate cancer, glioblastoma and astrocytoma) and appear to be involved in regulation of numerous immune genes, cell structure genes and cell signaling genes [225].

More recently, a study of serum samples from 33 patients with UTUC was conducted to identify biomarkers of tumor progression and cancer survival [226]. From a pool of 800 miRNAs that were evaluated, 38 were discovered to be differentially expressed in those patients who progressed compared to those whose UTUC did not progress. Independent validation of these 38 miRNAs in 21 UTUC patients (8 progressing and 13 non-progressing) refined the number of differentially expressed miRNAs to 18, of which miRNA-151b was a significant prognostic factor for tumor progression and cancer-specific survival [226]. They posit that the observed downregulation of miRNA-151b may impact downstream gene targets such as CCNE1 that is involved in T-cell signaling, DNA damage and cell cycle regulation, which has been shown to be altered in other cancer types such as breast, pancreas, lung and prostate cancers [226]. However, this is only one of 53 gene targets of miRNA-151b they identified in a network analysis of protein–protein interactions using two different software platforms. Thus, more work is clearly needed to map out and validate the underlying molecular implications of miRNA-151b downregulation in progressing UTUC.

While the precise causes and etiology of UTUC are not known, there are many associated risk factors that have been implicated which may contribute to disease onset [227]. These include geographic location, exposure to occupational and environmental toxins/chemicals, radiation therapy, bladder cancer, inflammation, tobacco smoke, as well as several other risk factors [227,228,229].

Along these lines, a recent study on the geographically restricted Balkan Endemic Neuropathy (BEN), which is strongly associated with UTUC, found a number of miRNAs that are differentially expressed in UTUC tissues of patients with UTUC that arose in BEN patients and in patients with non-BEN UTUC in comparison to normal kidney tissues [230]. Using miRNA expression data analyzed by two different statistical analysis software programs they identified miRNAs that result differentially expressed that were common to both programs for further analysis. In the BEN-UTUC patient group (*n* = 7) they found 10 differentially expressed miRNAs whereas in the non-BEN-UTUC patient group (*n* = 5) they found 15 differentially expressed miRNAs in comparison to normal kidney tissues. Of these, miRNA-205-5p was found to be upregulated in both BEN-UTUC and non-BEN-UTUC samples suggesting a role for this miRNA in the pathogenesis of UTUC [230]. Pathway analysis of miRNA-205-5p revealed potential gene targets, including extracellular matrix genes among others [230].

Tao et al. performed a miRNAs profiling of serum samples from 12 UTUC patients and 12 controls (cancer free patients of similar age); then, through RT-qPCR they validated 13 miRNAs as differentially expressed in serum samples of 46 UTUC patients compared to 30 controls [231]. Of these, statistical analysis revealed that miR-664a-3p, miR-431-5p, miR-423-5p, miR-191-5p, miR-33b-3p, miR-26a-5p, miR-22-3p, miR-16-5p, let-7b-5p and let-7c all had the potential to serve as diagnostic biomarkers of UTUC [231]. Notably, the expression levels of let-7b, let-7c and miR-22 have been shown to be altered in other types of cancers such as breast cancer, nasopharyngeal cancer, esophageal squamous cell carcinoma (ESCC) and non-small cell lung cancer (NSCLC) [231].

Finally, using tissue samples from 157 patients that had undergone radical nephroureterectomy, Browne et al. sought to determine if miRNA expression patterns could be used to stratify tumors by grade (high grade versus low grade) and whether the tumor invaded muscle [232]. They discovered that high-grade UTUC differentially expressed miR-29b-2-5p, miR-18a-5p, miR-223-3p and miR-199a-5p compared to low-grade UTUC [232]. Importantly, none of these were identified in the aforementioned studies. They also observed miRNAs differentially expressed in muscle-invasive compared with non-muscle-invasive samples: miR-10b-5p, miR-26a-5p-5p, miR-31-5p and miR-146b-5p [232]. Of these, miRNA-31 was previously reported [225]. The implications of this study are profound and, if confirmed, offer a level of fidelity to detection that could redefine staging and grading as well as open the door to development of novel targeted therapeutics.

All findings reported in this section are summarized in Table 1.

**Figure 1:**
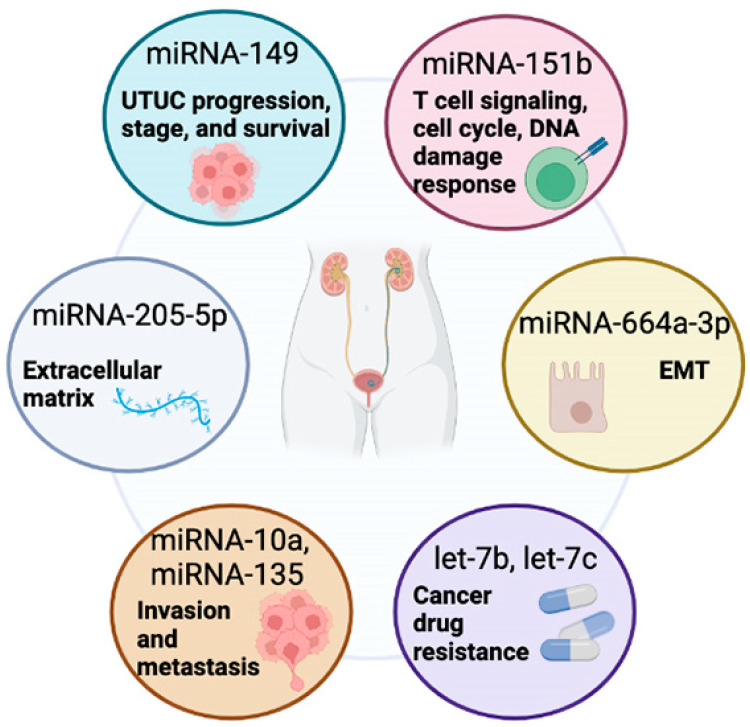
Diversity of miRNA Targets in UTUC. Examples of miRNAs identified in studies on UTUC biomarker screening. In most cases, the candidate miRNAs alter numerous genes and have pleotropic actions in normal and cancerous cells. The downstream actions presented represent some of the most common actions of these miRNAs.

The miRNAs identified in UTUC have many distinct molecular actions that need to be explored more carefully to better understand how they can be used clinically in diagnosis or to tailor therapeutics for management (Figure 1). A comprehensive summary of the main cited studies on the diagnostic and prognostic significance of miRNA in UTUC is listed in Table 1.

## 9. Conclusions

The biological characteristics of circulating as well as tissue miRNAs make them promising candidates as biomarkers of UTUC that could have diagnostic, prognostic and predictive utility. These circulating and tissue miRNAs may also represent potential therapeutic targets that could lead to development of novel therapeutic approaches.

## Figures and Tables

**Table 1:** miRNAs as diagnostic or prognostic biomarkers of UTUC.

Reference	Biomarker or Panel of Biomarkers and Levels	Sample Type	Collection Processing (Sample Processing, Storage Condition)	Study Design: Retrospective–Prospective; Monocenter-Multicenter	Discovery Cohort/Validation Cohort, Histotypes	Participant Characteristics, TNM Stage and Fuhrman Grade	Evaluation Method (RNA Extraction, Retrotrascription, and Detection Technology) and Normalization Strategy	Diagnostic/Prognostic Value and Statistical Analysis/Results
[194]	**Serum miRNAs.** ↑ miR-141 (UTUC patients vs. controls); ↓ miR-10a, ↓ miR135 in muscle-invasive UTUC (pTa/pT1 vs. pT2–4). **Tissue miRNAs.** ↑ miR-21, ↑ miR-96, ↑ miR-135, ↑ miR-141, ↑ miR-182, ↑ miR-205, ↑ miR-429, miR-520b, ↑ miR-10a, ↑ miR-200b (in UTUC tissue vs. normal tissue); ↑ miR-205 in undifferentiated UTUC (G3 vs. G2/G1).	Blood; Tissue samples	Pre-operative blood samples centrifuged at 2500× *g* × 10 min and stored in cryotubules at −80 °C until use; Formalin-fixed, paraffin-embedded tissue.	Prospective; multicenter (serum cohort), monocenter (tissue cohort)	**DC: Serum cohort:** 44 UTUC, 34 controls. **DC: Tissue cohort:** 47 UTUC, 36 controls.	**Serum cohort.** UTUC: Age: 67.9 y; Gender: 17 female; TNM: pTa/pT1 25 pts, pT2-pT4 19 pts; Grading: G1 6 pts, G2 28 pts, G3 10 pts. Control: Age: 63.5 y; Gender: 11 female; **Tissue cohort.** UTUC: Age: 68.9 y; Gender: 21 female; TNM: pTa/pT1 16 pts, pT2-pT4 31 pts; Grading: G1 9 pts, G2 20 pts, G3 18 pts. Control: Age: 69.1 y; Gender: 14 female;	Total RNA isolated from serum samples with mirVana PARIS Kit (Life Technologies) Total RNA extracted from tissues with the RecoverAll Total Nucleic Acid Isolation Kit (Life Technologies). Retrotrascription with miScript II RT Kit (Qiagen), qPCR using miScript SYBR Green PCR Kit (Qiagen). **Tissue**-miRNAs normalized against RNU1-4, SNORD43, and SNORD48, **serum**-miRNAs normalized against RNU1-4 and SNORD43	**Diagnostic.** Serum miR-141: AUC = 0.726 (70.5% sensitivity and 73.5% specificity) **Prognostic.** Serum miR-10a (*p* = 0.003) and miR-135 (*p* = 0.040)
[197]	↑miR-3648, ↑ miR-3656, ↑ miR-3687 (UTUC vs. normal kidney tissue)	Tissue samples	**DC:** Formalin-fixed, paraffin-embedded tissue samples **VC:** freshly frozen tissue samples	Retrospective, monocenter	**DC:** 24 ccRCC, 5 papRCC, 3 chRCC, 5 UTUC, 40 normal tissue samples **VC:** 10 ccrCC, 3 papRCC, 4 chRCC, 3 UTUC, 20 normal kidney samples	**DC:** ccRCC: Age: 70 y; Gender: 4 female; Stage: 13 T1, 6 T2, 5 T3, 0 T4, 2 metastatic samples; Grade: 0 G1, 12 G2, 9 G4, 3 G4. papRCC: Age: 67 y; Gender: 1 female; Stage: 4 T1, 1 T2, 0 T3, 0 T4; Grade: 0 G1, 2 G2, 3 G3, 0 G4. chRCC: Age: 68 y; Gender: 3 female; Stage: 0 T1, 3 T2, 0 T3, 0 T4, 0 metastatic; Grade: 0 G1, 1 G2, 2 G3, 0 G4. UTUC: Age: 76 y; Gender: 1 female; Stage: 3 T1, 1 T2, 1 T3, 0 T4, 0 metastatic; Grade: 0 G1, 1 G2, 3 G3, 0 G4.	Total RNA extracted from FFPE tissue samples with High Pure FFPE RNA Micro Kit (Roche Applied Science); Total RNA extracted from frozen tissue samples with mirVANA miRNA Isolation Kit (Ambion). MicroRNA microarray analysis with miRCURY LNA microRNA Array, 6th gen (Exiqon). Retrotranscription using miRCURY LNA Universal RT cDNA synthesis kit (Exiqon). qPCR using SYBR Green master mix (Exiqon, Vedbaek, Denmark) and LNA microRNA-specific primers on a ViiA 7 Real-Time PCR System (Applied Biosystems) and SNORA66, U6snRNA, RNU1A1 as endogenous reference genes for normalization.	**Diagnostic.** miR-3648, miR-3656, miR-3687 discriminated UTUC from normal kidney tissue with a median AUC > 0.94, *p* < 0.001
[199]	↑ miR-488, ↓ miR-181c (AAN-UTUC vs. non-AAN-UTUC); ↑ miR-488 (stage I-II vs. stage III-IV AAN-UTUC); ↑ miR-181 (>3 cm vs. <3 cm AAN-UUC)	Tissue samples	Formalin-fixed, paraffin-embedded tissue samples	Retrospective, Monocenter	20 cases of AAN-UTUC and 20 controls (non AAN-UTUC)	**AAN-group:** Age: 63.9 ± 1.64 y; Gender: 11 female; Stage: 9 stage I–II, 11 stage III–IV; Tumor differentiation: 4 well, 7 moderate, 9 poor. **Non AAN-group:** Age: 65.6 ± 1.66 y; Gender: 7 female; Stage: 7 stage I–II, 13 stage III–IV; Tumor differentiation: 9 well, 5 moderate, 6 poor.	RNA isolation using mirVanaTM miRNA isolation kit (Life Technologies); miRNA microarray analysis using Affymetrix GeneChip miRNA arrays (Affymetrix); reverse transcription using a stem-loop RT primer; qPCR reaction with TaqMan PCR Master Mix-UNG using an ABI 7900 HT real-time PCR system (Applied Biosystems).	**Diagnostic.** miR-488 and miR-181c: AAN-UTUC vs. non-AAN-UTUC (*p* < 0.05); **Prognostic.** miR-488: stage I–II vs. stage III–IV AAN-UUC (*p* = 0.038); miR-181c: >3 cm vs. <3 cm AAN-UUC (*p* = 0.049).
[203]	↑ miR-210 (UTUC tissue vs. normal tissue); high-stage and high-grade tumors vs. low-stage and low-grade tumors)	Tissue samples	Freshly frozen tissue samples	Retrospective, monocenter	A total of 83 surgically removed UTUC cases provided 83 **UTUC** tissue samples and 50 paired **normal urothelium** tissue samples	**All cohort:** Age: 42 pts <70 y and 41 pts ≥70 y pts; Gender: 48 females; Stage: 50 organ-confined (T1–T2), 33 locally advanced (T3–T4); Grade: 11 Low, 72 High	Total RNA extracted with Quick-RNA™ MiniPrep Kit (Zymo research, Reverse-transcription using miR-210-specific stem-loop primer; qPCR using TaqMan miRNA assay (Applied Biosystems) on 7500HT Fast Real Time PCR System (Applied Biosystems). RNU6B as endogenous reference gene for normalization.	**Diagnostic.** UTUC from non-cancerous Urothelium with an AUC of 0.904 (95% CI = 0.843–0.865, *p* < 0.001), with 80% sensitivity and 90% specificity; **Prognostic:** high stage vs. low stage, *p* = 0.02; high grade vs. low grade, *p* = 0.049
[208]	↓ miR-145-5p (UTUC vs. normal urothelium tissues)	Tissue samples	Formalin-fixed, paraffin-embedded tissue samples	Retrospective, monocenter	114 UTUC samples and 40 paired non tumoral urothelium samples. Subsequent focal analysis of miR-145-5p was restricted to 61 pairs of UTUC samples and normal tumor-adjacent tissue samples	**DC.** Age: 41 pts <65y, 73 pts ≥65y; Gender: 65 females; Stage: 75 T1–T2, 39 T3–T4; Grade: 24 low, 90 high.	Total RNA extracted with TRIzol reagent (Invitrogen); reverse transcription with TaqMan MicroRNA Reverse Transcription Kit (Applied Biosystem). qPCR using TaqMan MicroRNA Assays on StepOnePlus Real-Time PCR System (Applied Biosystems). U6 as endogenous reference gene for normalization.	**Diagnosis.** UTUC vs. normal urothelium tissues (*p* < 0.001).
[222]	↓ miR-30a-5p (UTUC tissues vs. normal tissues)	Tissue samples	Fresh tissues were immediately immersed in RNA later (Qiagen) after nephroureterectomy, stored at 4 °C overnight, and then stored at −80 °C	Retrospective, monocenter	22 UTUC pts (renal pelvis) who underwent nephroureterectomy without neoadjuvant therapy provided 22 UTUC tissue samples and 14 Adjacent non-tumoral tissue collected from non-cancer areas (by gross appearance) of renal pelvis mucosa	**DC.** Age: 4 < 60y, 18 ≥ 60 y; Gender: 13 female; Stage: 12 T1-T2, 10 T3-T4, 15 N0, 7 N1, 9 M0, 13 M1; Grade: 5 Low, 17 High.	Total RNA extracted with TRIzol Reagent (Invitrogen); reverse transcription with TaqMan MicroRNA reverse transcription kit (ABI); qPCR with TaqMan microRNA assays using Brilliant III QPCR Master Mixes with low ROX on AriaMx Real-Time PCR system (Agilent). U6 as endogenous reference gene for normalization.	miR-30a-5p is significantly downregulated in UTUC samples (*p* < 0.001).
[225]	↑ miRNA-31,↑ miRNA-149 (non-progressing UTUC vs. progressing UTUC	Tissue samples	Formalin-fixed paraffin-embedded tissue samples	Retrospective, multicenter	A total of 150 patients with RNU-treated UTUC	**Total cohort:** Age: 70 y; Gender: 34 female; Stage: 26 pTa, 42 pT1, 28 pT2, 38 pT3, 16 pT4; Grade: 14 G1, 48 G2, 88 G3. **DC:** 18 randomly selected UTUC cases (9 non-progressing UTUC and 9 progressing UTUC) **VC:** remaining 132 UTUC pts	Total RNA isolation using the RecoverAll Total Nucleic Acid Isolation Kit (Ambion). **Screening Phase:** Reverse transcription with TaqMan miRNA reverse transcription kit with Megaplex RT primers for Human Pool A and B (Applied Biosystems). Pre-Amplification with Megaplex PreAmp primers (Applied Biosystems); qPCR with TaqMan^®^ Human MicroRNA Array A + B Cards Set v2.0 on ABI7900HT Real-Time PCR system (Applied Biosystems) and global mean as normalization method. **Validation phases:** RT-qPCR with miRCURY Locked Nucleic Acid kit (Exiqon) on ABI7900HT Real-Time PCR system (Applied Biosystems). hsa-miR-218 as endogenous reference gene for normalization.	**Prognosis.** miR-31 and miR-149 were independent prognostic factors of tumor progression (HR 0.88, *p* < 0.001 and HR 0.78, *p* < 0.001, respectively); miR-149 expression was an independent prognostic factor of cancer-specific survival (HR 0.82; *p* = 0.018).
[226]	↓ miR-151b (Progressing vs. non-progressing UTUC pts)	Serum samples	Pre-operative blood samples collected in a BD vacutainer sterile tube coated with silicone and micronized silica particles, left to clot for a minimum of one hour and, within four hours, centrifuged for 15 min at 3500 rpm at 4 °C; serum transferred to a cryotube and stored at −80 °C.	Prospective, monocenter	A total of 33 pts with UTUC were analyzed in two phases, DC. 5 progressing and 7 non-progressing. VC: 8 progressing and 13 non-progressing UTUC pts.	**Total cohort.** Age: 70 y; Gender: 10 female; Stage: 7 pTa, 5 pT1, 7 pT2, 12 pT3, 2 pT4; Grade: 6 Low, 27 High. **DC:** progressing pts: Stage and Grade: 1 pT2, 3 pT3 and 1 pT4, all high grade; non-progressing: Stage and Grade: 2 pTa, 1 pT2 and 4 pT3, all high-grade. VC: progressing pts: Stage and Grade: 1 pTa low grade, 1 pT1 low-grade, 2 pT2 high grade, 3 pT3 high grade and 1 pT4 high grade; non-progressing: Stage and Grade: 4 pTa low grade, 4 pT1 high grade, 3 pT2 high grade and 2 pT3 high grade).	Total RNA extracted with mirVana PARIS Kit (Thermosfisher Scientific), miRNAs expression profiling using nCounter Human v3 miRNA expression Assay Kit (NanoString Technologies). hsa-miR-16-5p, hsa-miR-484, hsa-miR-126-3p, hsa-miR-191-5p, hsa-miR-93-5p and hsa-miR-24-3p as endogenous reference genes for normalization.	**Prognosis.** miR-151b prognostic factors for tumor progression (HR = 0.33, *p* < 0.001) and cancer specific survival (HR = 0.25, *p* < 0.001).
[230]	*↑hsa-miR-205-5p*, ↑ hsa-miR-4322, ↑ hsa-miR-99b-3p, ↑ hsa-miR-3620-3p, ↑ hsa-miR-373-5p, ↑ hsa-miR-3656, ↑ hsa-miR-1290, ↓ hsa-miR-30a-5p, ↓ hsa-miR-127-3p and ↓ hsa-miR-154-5p in BEN-UTUC vs. normal kidney tissue; *↑* *hsa-miR-205-5p*, ↑ hsa-miR-205-3p, ↑ has-miR-224-5p, ↑ hsa-miR-224-3p, ↑ hsa-miR-197-3p, ↑ hsa-miR-182-5p, ↑ hsa-miR-183-5p, ↑ hsa-miR-96-5p, ↑ hsa-miR-203a-3p, ↑ hsa-miR-149-5p, ↑ hsa-miR-141-3p, ↑ hsa-miR-200c-3p, ↑ hsa-miR-1260a, ↑ hsa-miR-210-3p, ↓ hsa-miR-663b in non-BEN-UTUC vs. normal kidney tissue	Tissue samples	Formalin-fixed paraffine embedded tissue samples	Retrospective, monocenter	7 BEN-UTUC, 5 non-BEN-UTUC and 8 normal kidney tissues.	**DC.** BEN-UTUC: Age: 66 y; Gender: 2 female; Stage: 4 Low, 2 high, 1 unknown; Grade: 4 low, 2 high, 1 unknown; Non-BEN-UTUC: Age: 62.8 y; Gender: 3 female; Stage: 3 low, 2 high; Grade: 1 low, 4 high. Normal kidney tissue samples: unspecified clinical–pathological features.	Total RNA extraction with FFPE DNA/RNA Kit (Qiagen). **miRNAs expression profiling:** “miRNA Microarray System with miRNA Complete Labeling and Hyb Kit,” version 2.4 with Agilent Sure Print G3 Human v16 miRNA Array Kit (Agilent Technologies); **RT-qPCR Validation:** reverse transcription with TaqMan_ MicroRNA Reverse Transcription Kit (Life Technologies); qPCR with TaqMan Universal PCR Master Mix and TaqMAN MicroRNA Assays (Life Technologies).	**Diagnostic.** BEN-UTUC vs. normal kidney tissue, *p* < 0.05; non-BEN-UTUC vs. normal kidney tissue, *p* < 0.05.
[231]	↑ miR-664a-3p ↑ miR-431-5p ↑ miR-423-5p ↑ miR-191-5p ↑ miR-33b-3p ↑ miR-26a-5p ↑ miR-22-3p ↑ miR-16-5p ↑ let-7b-5p ↑ let-7c (UTUC patients vs. controls)	Serum samples	Blood drawn within 1 day after admission, the coagulated blood samples were collected in tubes containing a separating gel and clot activator, then centrifugated at 1500× *g* for 15 min at 4 °C. The supernatant was centrifugated at 1500× *g* for 15 min and stored at −80 °C.	Retrospective, monocenter	58 UTUC patients and 42 cancer-free controls with non-neoplastic hematuria (12 UTUC pts and 12 controls in the training set, 46 UTUC pts and 30 controls in the validation set)	**DC.** UTUC: Age: 28 <68 y, 30 ≥68 y; Gender: 17 female; Stage: 21 I stage, 14 II stage, 20 III stage, 3 stage IV; Grade: 1 papillary urothelial neoplasia of low malignant potential, 17 low, 40 high. Normal: Age: 22 <60 y, 20 ≥68 y; Gender: 12 female.	RNA extracted with miRNeasy Serum/Plasma kit (Qiagen); miRNAs expression profiling: deep sequencing platform Illumina HiSeq™ 2000; RT-qPCR Validation: All-in-One miRNA RT-qPCR Detection kit (GeneCapoiea-FulenGen). qPCR with SYBR green on Applied Biosystems Step One Plus System (Applied Biosystems). RNU6-2 as endogenous reference gene for normalization.	**Diagnosis.** UTUC patients vs. controls, AUC > 0.8
[232]	↓ hsa-miR-10a-5p, ↓ hsa-miR-10b-5p, ↓ hsa-miR-26a-5p, ↓ hsa-miR-29b-2-5p, ↓ hsa-miR-31-5p, ↑ hsa-miR-146b-5p, ↑ hsa-miR-223-3p (G3–G4 vs. G1–G2 and ≥ pT2 vs. pTa/pT1); ↑ hsa-miR-18a-5p and ↓ hsa-miR-199a-5p (G3–G4 vs. G1–G2); hsa-miR-30c-5p (≥pT2 vs. pTa/pT1).	Tissue samples	Formalin fixed paraffin embedded tissue samples	Retrospective, multicenter	157 patients with UTUC (35 constituted the screening cohort while 123 constituted the validation cohort)	**DC:** Age: 73.2 y; Gender: 49% female; Stage: 16 pTa, 3 pT1, 4 pT2, 10 pT3, 3 pT4; Grade: 42.9% low, 57.1% high. **VC:** 70.9 y; Gender: 33%; Stage: 45 pTa, 31 pT1, 15 pT2, 28 pT3, 4 pT4; Grade: 37% low, 63% high.	Total RNA extracted with RecoverAll^®^ Total Nucleic Acid Isolation Kit (Ambion). RT-qPCR using miRCURY LNA™ reagents (Exiqon). Screening analysis with Human miRNome panels I and II version 4, analyzing 752 miRNA (Exiqon), using global mean as normalization method. RT-qPCR Validation using miRNA LNA primer sets.	**Prognostic.** G3–G4 vs. G1–G2: miR-29b-2-5p, miR-18a-5p, miR-223-3p and miR-199a-5p, with AUC = 0.86 (83% sensitivity, 85% specificity); ≥pT2 vs. pTa/pT1: miR-10b-5p, miR-26a-5p-5p, miR-31-5p and miR-146b-5p, with AUC = 0.9 (64% sensitivity, 96% specificity).

UTUC—upper tract urothelial cancer; miRNA—microRNA; DC—discovery cohort; VC—validation cohort; pts—patients; RT-qPCR—Reverse Transcription quantitative PCR; RNU1-4—U1 Small Nuclear 4 RNA; SNORD43—Small Nucleolar RNA, C/D Box 43; SNORD48—Small Nucleolar RNA, C/D Box 48; AUC—area under the curve; ccRCC—clear cell Renal Cell Carcinoma; papRCC—papillary Renal Cell Carcinoma; FFPE—formalin-fixed paraffin-embedded; SNORA66—Small Nucleolar RNA, H/ACA Box 66; U6snRNA—U6 spliceosomal RNA; RNU1A1—U1 Small Nuclear 1 RNA; AAN-UTUC—Aristolochic Acid-induced upper tract urothelial cancer; RNU6B—U6B small nucleolar RNA; BEN-UTUC—Upper Tract Urothelial Cancer patients living in endemic regions for Balkan endemic Nephropathy; U6 Small Nuclear 2 RNA).
